# Characteristics and Outcomes of T1a Renal Cell Carcinoma Presenting with Metastasis

**DOI:** 10.3390/cancers17030364

**Published:** 2025-01-23

**Authors:** Luke Wang, Melis Guer, Dhruv Puri, Franklin Liu, Sohail Dhanji, Margaret F. Meagher, Aastha Shah, Saeed Ghassemzadeh, Juan Javier-DesLoges, James Brugarolas, Payal Kapur, Aditya Bagrodia, Brent Rose, James D. Murphy, Ithaar H. Derweesh, Rana R. McKay

**Affiliations:** 1 Department of Urology, School of Medicine, University of California San Diego, La Jolla, CA 92093, USA; 2 Clinic of Urology, Uro-Oncology, Robot-Assisted and Focal Therapy, University Hospital Magdeburg, 39120 Magdeburg, Germany; 3 Department of Medicine, College of Medicine, University of Arizona, Tuscon, AZ 85724, USA; 4 Department of Urology, Health Science Center, University of Tennessee, Memphis, TN 38163, USA; 5 Department of Internal Medicine, UT Southwestern Medical Center, Dallas, TX 75390, USA; 6 Moores UCSD Cancer Center, School of Medicine, University of California San Diego, La Jolla, CA 92037, USA; 7 Department of Radiation Medicine and Applied Sciences, University of California San Diego, La Jolla, CA 92093, USA; 8 Division of Hematology-Oncology, Department of Medicine, School of Medicine, University of California San Diego, La Jolla, CA 92093, USA

**Keywords:** carcinoma, renal cell, T1a, national cancer database, nephrectomy, synchronous metastasis, survival analysis

## Abstract

Objectives: The incidence of renal cell carcinoma (RCC) has been rising, largely due to increased incidental detection from widespread imaging. Although synchronous distant metastasis (SM) with a primary renal tumor measuring <4 cm (cT1a) is uncommon, its presence may influence survival outcomes and the utility of cytoreductive nephrectomy. We sought to investigate clinical characteristics, metastatic patterns, treatments, and survival outcomes of patients with T1a RCC. Methods: All patients aged ≥18 years diagnosed with RCC between 2004 and 2019 were extracted from the National Cancer Database. The Cochran–Armitage test was used for trend analysis, while multivariable analyses were conducted to identify variables associated with SM and to assess the impact of cytoreductive surgery on mortality across isolated metastatic sites. Kaplan–Meier analysis was performed to compare survival outcomes. Results: A total of 263,911 individuals diagnosed with T1a RCC were analyzed in the study. Among them, 114,661 patients (43.4%) were classified as having cT1a tumor stage, and of these patients with cT1a RCC, 2275 (2.0%) exhibited SM. The proportion of SM cT1a was 3.39% in 2004 and 2.08% in 2019, with an Average Annual Percent Change (AAPC) of −0.037% (*p* = 0.830). The most common sites of metastasis were bone (59%), followed by lung (35%), liver (16%), and brain (12%). Resection of the primary tumor and receipt of systemic therapy were significantly associated with reduced mortality among all metastatic sites, especially in individuals with lung-only metastases (HR = 0.02, *p* = 0.013). Metastasectomy was associated with improved survival in patients with brain-only metastases (HR = 0.26, *p* = 0.006) but did not demonstrate the same benefit in patients with bone-, lung- or liver-only metastases. The worst 5-year OS rate was observed in cases with metastasis to multiple sites, whereas isolated metastases had similar survival rates (*p* < 0.0001). Our findings are limited by retrospective study design. Conclusions: This comprehensive analysis of T1a RCC patients reveals that while synchronous metastasis is relatively uncommon (2.0%), it presents significant clinical challenges, with bone as the most common metastatic site, contrasting with the typical lung predominance in larger tumors. Primary tumor resection showed survival benefit in patients with isolated metastases, especially for lung-only metastasis. These findings highlight the heterogeneous nature of tumor biology in small renal masses and underscore the importance of tailored, multimodal treatment strategies for the effective management of SM T1a RCC.

## Simple Summary

While the presentation of synchronous distant metastasis (SM) with primary renal cell carcinoma (RCC) measuring <4 cm (T1a) is uncommon, its impact on survival outcomes in the era of modern treatment strategies remains unknown. This study aims to evaluate the temporal trends in the prevalence of SM in T1a RCC, with a focus on variations in clinicopathologic characteristics, identification of variables associated with SM, patterns of metastases, and survival outcomes. Utilizing the National Cancer Database, 263,911 patients were included in the analysis. Bone was the most common metastatic site, contrasting with the lung predominance in larger tumors. Primary tumor resection demonstrated survival benefit in patients with isolated metastases, especially those with lung-only metastases. These findings highlight the heterogeneous nature of tumor biology in small renal masses and underscore the importance of tailored, multimodal treatment strategies for the effective management of SM T1a RCC.

## 1. Introduction

The incidence of renal cell carcinoma (RCC) has been increasing, largely as a result of the widespread use of imaging and increased incidental detection of primary tumors [1,2]. Patients with primary tumors ≤ 4 cm (T1a) without metastasis are defined as Stage I RCC in the American Joint Committee on Cancer (AJCC) staging system, with an estimated 5-year survival rate of 97%. For patients who present with de novo synchronous metastatic disease, survival rates decrease to 17% at 5 years [2]. Notably, the timing of systemic therapy initiation relative to diagnosis is a key prognostic factor in the International Metastatic RCC Database Consortium (IMDC) model, with initiation less than 1 year from diagnosis associated with poorer outcomes [3]. In the last decade, immunotherapy (IO)-based combination therapies have shown a significant survival benefit for patients with metastatic disease [4]. While the presentation of synchronous distant metastasis (SM) with a primary renal tumor measuring < 4 cm (T1a) is uncommon, its impact on survival outcomes in the era of modern treatment strategies remains unknown [5]. Identifying T1a RCC with SM represents a clinical challenge, as the tumor characteristics remain poorly understood, and the integration of multimodal treatment strategies with systemic therapy and primary tumor-directed treatments, including surgery, has not been well established [6]. Further research is required to undercover the underlying complex nature of SM T1a RCC to develop advanced diagnostic and treatment strategies.

The survival benefit of cytoreductive surgery in the context of SM RCC has been widely debated [7]. Results from CARMENA and SURTIME trials have questioned the role and optimal sequence of cytoreduction in the era of targeted therapies [8,9]. Thus, the majority of study cohorts had advanced local tumor stages. Considering the results of the CARMENA trial, which reported a median tumor size of 8.6–8.8 cm and only 7.5% of the study cohort classified as T1 stage, the CARMENA trial may exhibit bias regarding clear conclusions for patients with small primary tumors.

This study aims to evaluate the temporal trends in the prevalence of T1a RCC with SM, focusing on variations in clinicopathologic characteristics between clinical (c) and pathological (p) T1a RCC with SM and identifying variables associated with SM, patterns of metastases, and survival outcomes. This is important for understanding the underlying complexity of metastasis development in the context of low-stage primary disease and improving patient selection for multimodal approaches. Given the paucity of data on the outcomes of patients with T1a RCC with SM, we utilized the National Cancer Database (NCDB), which offers a reliable and accessible data source for comprehensive analysis in RCC to dissect the characteristics and outcomes of patients.

## 2. Methods

### 2.1. Patient Population and Study Design

We analyzed the National Cancer Database (NCDB), which comprises data from over 1500 hospitals accredited by the Commission on Cancer (CoC) and accounts for 70% of new cancer diagnoses in the United States [10]. The study population consists of adults ≥ 18 years of age diagnosed from 2004 to 2019 with RCC. RCC was classified as presence of *International Classification of Diseases for Oncology—Third Edition*, code C64.9 [11]. We included patients with cT1a and pT1a tumor stages, restricting the selection to those with a single malignant primary tumor to minimize confounding from multiple independent malignancies [11]. Moreover, we omitted patients with unknown metastasis status, metachronous metastasis, and those treated outside the reporting facility. SM was defined in patients with cT1a or pT1a RCC as those coded with AJCC prognostic stage IV at diagnosis or with metastasis recorded at the initial diagnosis, irrespective of the site. Then, we allocated patients into the cohorts: cT1a with/without SM and pT1a with/without SM. There were no missing data regarding the presence of SM. A flow diagram of patient distribution is presented in Supplementary Figure S1. Informed consent from patients and Institutional Review Board approval were not required, as the data used in the study are readily available in the mentioned repositories in a de-identified format.

### 2.2. Data Collection

Demographic and clinical characteristics included patient age, sex, race, ethnicity, year of diagnosis, facility location categorized by Facility Oncology Registry Data Standards (FORDS) based on United States Census Divisions, facility type, median household income, Charlson comorbidity index, tumor size, and clinical N stage, SM at any site (from 2004 to 2019), as well as specifically including lung, bone, liver, and brain (from 2010 to 2019) [12]. According to NCDB, race and ethnicity are based on chart reviews of patients’ self-reported demographics [11]. Pathologic characteristics included tumor histology, tumor grade, presence of sarcomatoid dedifferentiation, tumor necrosis, and lymphovascular invasion. Treatment data included surgery of the primary tumor site, margin status, surgery of lymph nodes or tissues/organs beyond the primary site (metastasectomy), and receipt of systemic therapy. Additional information about selected variables can be found in the NCDB 2019 Participant User File [11].

### 2.3. Statistical Analysis

Trend analyses of SM cT1a and pT1a RCC were performed using the Cochran–Armitage test. The average annual percentage change (AAPC) was calculated by applying linear regression to the computed percentages. Locally estimated scatterplot smoothing was used to analyze trends from 2004 to 2019 in the proportion of SM cT1a and pT1a RCC. Multivariable logistic regression was performed to calculate the odds ratio (OR) and determine variables associated with SM in cT1a and pT1a RCC. Multivariable Cox-proportional-hazards regression was used to estimate the hazard ratio (HR) and analyze the impact of demographic, clinical, and pathologic characteristics on overall survival (OS) in SM cT1a and pT1a RCC. For the sub-analyses evaluating the impact of cytoreductive surgery on all-cause mortality (ACM), the cT1a cohort was selected, as this model is intended for the decision-making process in the preoperative setting. Kaplan–Meier analysis was utilized to compare survival outcomes. Data were managed on SPSS Version 21 (IBM Corporation, Armonk, NY, USA), and all statistical analyses were conducted on R Studio (R Studio Team, Boston, MA, USA). Clear cell histology was designated as reference histology for all analyses. Holm correction was applied to all categorical variables with more than two levels reported in the multivariable models. Two-tailed *p* values < 0.05 were considered statistically significant.

## 3. Results

### 3.1. Study Cohort and Temporal Trend Analyses

Overall, a total of 263,911 patients with RCC from the NCDB were included in the analysis. Of these, 114,661 patients (43.4%) had cT1a tumor stage, and within this group, 2275 (2.0%) presented with SM. The pT1a tumor stage was observed in 115,078 patients (43.6%). Of these, 440 (0.4%) presented with SM. Among SM cT1a (*n* = 2275), only 265 (11.6%) patients were diagnosed with pT1a following surgery. Venn diagrams (Supplementary Figure S2) were used to illustrate case overlap between cT1a and pT1a RCC (with/without SM).

**Figure 1:**
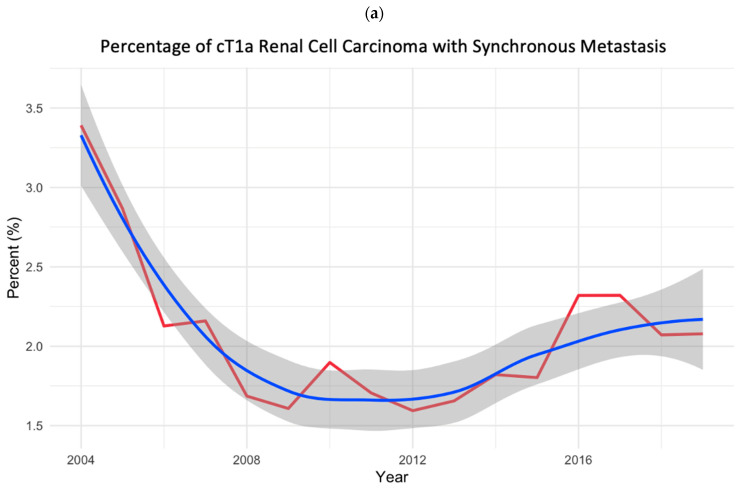

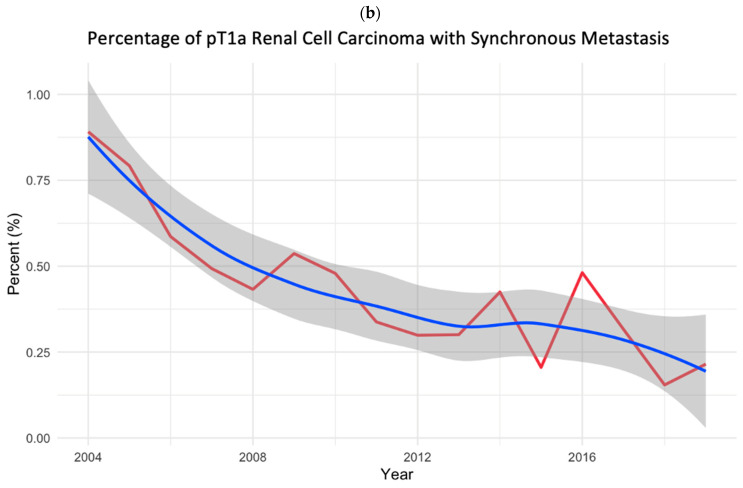
Trend analyses of changes in percentage of (**a**) cT1a RCC with synchronous metastasis among all cases of cT1a RCC and (**b**) pT1a RCC with synchronous metastasis among all cases of pT1a RCC diagnosed from 2004 to 2019. Red line indicates annual percentages. Blue line indicates trend line fitted using locally estimated scatterplot smoothing. Gray indicates 95% confidence intervals.

Over the study period, the proportion of SM cT1aRCC decreased from 3.3% in 2004 to 2.0% in 2019, with an AAPC of −0.037% (Cochran–Armitage *p* = 0.830). The proportion of SM pT1aRCC decreased from 0.8% in 2004 to 0.2% in 2019 with an AAPC of −0.036% (Cochran–Armitage *p* < 0.001) (Figure 1).

### 3.2. Patient Characteristics

Cohort demographics according to clinical or pathological T1a and M status are summarized in Supplementary Table S1. Both subgroups with SM (cT1a and pT1a RCC) were significantly older (*p* < 0.001), predominantly male (*p* < 0.001) with larger tumor size (*p* < 0.001), higher tumor grade (*p* < 0.001), and sarcomatoid dedifferentiation (*p* < 0.001) compared to those without SM.

The median follow-up of SM cT1aRCC was 8.8 months [interquartile range (IQR) 2.6–27.0]. The most common site of metastasis was bone (59%), followed by lung (35%), liver (16%), and brain (12%). Of these cT1a patients, 11% underwent radical nephrectomy (*n* = 255) and 4.7% had partial nephrectomy (*n* = 106), resulting in pT1a post-surgery in 265 overlapping cases. Overall, 45.2% of patients with cT1a received systemic therapy (*n* = 1029).

In the SM pT1aRCC group, the median follow-up was 35.3 months (IQR 16.1–68.5), with bone as the most common site of SM (64%), followed by lung (21%), brain (6.1%), and liver (4.7%). All patients with SM pT1aRCC had surgery for the primary tumor (73% radical nephrectomy/27% partial nephrectomy), and 37% received systemic therapy.

### 3.3. Variables Associated with Synchronous Metastasis

In the multivariable logistic regression model (Table 1), SM in cT1a RCC was associated with increasing age (OR = 1.02, *p* < 0.001), male sex (OR = 1.49, *p* < 0.001), larger tumor size (OR = 1.7, *p* < 0.001), cN1 (OR = 319.0, *p* < 0.001), collecting duct histology (OR = 11.9, *p* < 0.001), medullary histology (OR = 73.7, *p* < 0.001), sarcomatoid dedifferentiation (OR = 2.41, *p* < 0.001), and tumor grades 3–4 (OR = 4.08–12.2, *p* < 0.001). Conversely, patients diagnosed at an academic center or integrated cancer network (OR = 0.62–0.64, *p* < 0.001), with income in the fourth (highest) quartile (OR = 0.7, *p* < 0.001) and with papillary (OR = 0.39, *p* < 0.001) and chromophobe (OR = 0.09, *p* < 0.001) histology were less likely to have SM.

For SM in pT1a RCC, independent predictors included increasing age (OR = 1.01, *p* = 0.002), male sex (OR = 1.61, *p* < 0.001), larger tumor size (OR = 1.79, *p* < 0.001), cN1 (OR = 95.3, *p* < 0.001), sarcomatoid dedifferentiation (OR = 5.41, *p* < 0.001), and increasing tumor grade (OR = 1.73–16.7, *p* < 0.001). Patients diagnosed between 2010 and 2015 (OR = 0.75, *p* = 0.042) and 2016 and 2019 (OR = 0.58, *p* < 0.001), those with Charlson score ≥ 2 (OR = 0.69, *p* = 0.047), and those with papillary (OR = 0.4, *p* < 0.001) and chromophobe (OR = 0.32, *p* = 0.002) histology were less likely to exhibit SM.

### 3.4. Variables Associated with All-Cause Mortality

The results of multivariable Cox regression analysis for all-cause mortality (ACM) are listed in Table 2. In the SM cT1aRCC group, ACM was associated with increasing age (OR = 1.01, *p* < 0.001), Charlson ≥ 2 (OR = 1.28, *p* = 0.004), cN1 (OR = 1.61, *p* < 0.001), metastasis to multiple organs (OR = 1.77, *p* < 0.001), sarcomatoid dedifferentiation (OR = 2.04, *p* < 0.001), positive margin (OR = 2.07, *p* = 0.028). Conversely, decreased risk of ACM was observed in patients with diagnosis at academic center (OR = 0.66, *p* = 0.003) and those who received systemic therapy (OR = 0.49, *p* < 0.001). Among the SM pT1aRCC, ACM was associated with increasing age (OR = 1.03, *p* = 0.005) and cN1 (OR = 3.37, *p* = 0.021).

Table 3 and Table 4 show the exploratory multivariable Cox regression model evaluating the impact of cytoreductive surgery on ACM in cT1a RCC with SM to bone, lung, liver, and brain. This subgroup analysis was conducted in cT1aRCC, as this is the information available at the time of treatment decision at the initial diagnosis. First, in patients with SM bone-only, worsened ACM was associated with increasing age (HR = 1.03, *p* < 0.001), Charlson ≥ 2 (HR = 1.56, *p* = 0.019), cN1 (HR = 1.91, *p* < 0.001), medullary histology (HR = 10.2, *p* = 0.004), and sarcomatoid dedifferentiation (HR = 2.05, *p* = 0.039). A lower risk of ACM was observed in patients who had undergone partial nephrectomy (HR = 0.23, *p* = 0.011) and received systemic therapy (HR = 0.57, *p* < 0.001).

Second, independent risk factors of worsened ACM in patients with SM lung-only were Charlson ≥ 2 (HR = 2.37, *p* = 0.004) and sarcomatoid dedifferentiation (HR = 4.09, *p* = 0.013), while decreased risk of ACM was observed in patients undergone nephrectomy (HR = 0.08, *p* = 0.047) and partial nephrectomy (HR = 0.02, *p* = 0.013), and received systemic therapy (HR = 0.36, *p* < 0.001). Third, in patients with liver-only SM, worsened ACM was associated with larger primary tumor size (HR = 1.7, *p* = 0.010). Patients who had undergone nephrectomy (HR = 0.22, *p* = 0.039) and received systemic therapy (HR = 0.41, *p* = 0.033) demonstrated a lower risk of ACM. Fourth, in brain-only SM, worsened ACM was observed in patients with larger tumor size (HR = 2.44, *p* = 0.006). A lower risk of ACM was associated with nephrectomy (HR = 0.08, *p* = 0.005), partial nephrectomy (HR = 0.1, *p* = 0.047), metastasectomy (HR = 0.26, *p* = 0.006), and systemic therapy (HR = 0.22, *p* < 0.001).

### 3.5. Kaplan–Meier Analysis of Overall Survival

**Figure 2:**
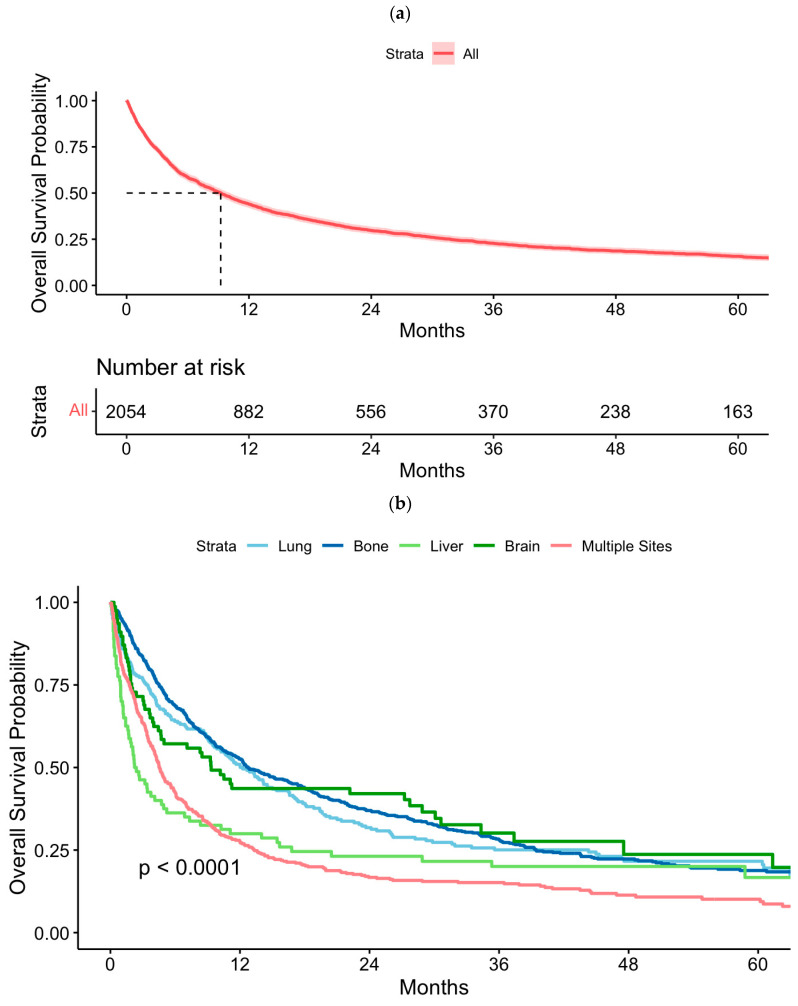

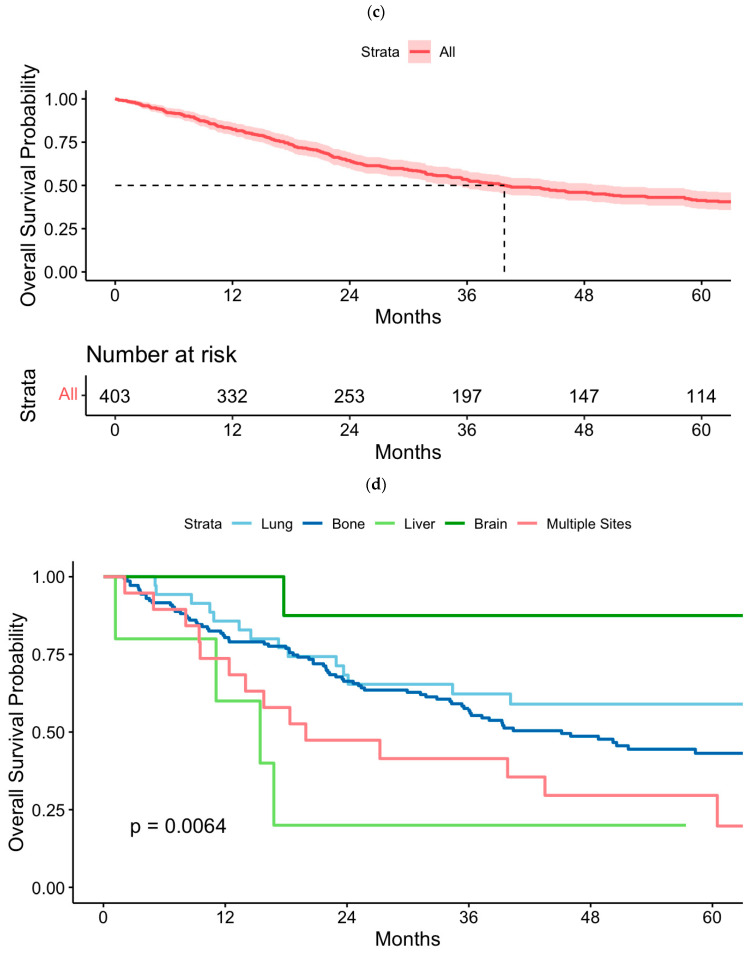
Overall survival in (**a**) cT1a renal cell carcinoma (RCC) with synchronous metastasis, (**b**) cT1a RCC with synchronous metastasis stratified by organ site, (**c**) pT1a RCC with synchronous metastasis, (**d**) pT1a RCC with synchronous metastasis stratified by organ site.

Figure 2a,c show overall survival (OS) in SM cT1a and pT1a RCC. Figure 2b,d display OS stratified by lung-only, bone-only, liver-only, brain-only, and multiple metastatic sites in cT1a and pT1a RCC, respectively. Five-year OS in patients SM cT1a RCC was 15.8% (95% CI 14.1–17.6), while for SM pT1a RCC was 41.3% (95% CI 36.5–46.7). In the SM cT1aRCC group, the worst 5-year OS rate was observed in cases with metastasis to multiple sites, whereas bone-only, lung-only, liver-only, and brain-only metastasis had similar survival rates (*p* < 0.0001). In the SM pT1a group, the worst 5-year OS was observed in cases with metastasis to the liver-only and to multiple sites, followed by bone-only metastasis, though interpretation is limited due to the small sample size (*p* = 0.0064).

## 4. Discussion

We present a comprehensive analysis of trends, clinical characteristics, and survival outcomes of SM T1a RCC in a large cohort of patients in the NCDB and provide the following noteworthy results. First, we observed a declining trend in the annual percentage of SM in both cT1a and pT1a RCC from 2004 to 2019. Second, for both SM cT1a and pT1a, bone was the most frequent site of SM. Third, the predictors of SM cT1a RCC included increasing age, male sex, higher tumor grade, histology of medullary RCC, collecting duct, and sarcomatoid dedifferentiation. Fourth, primary tumor resection and systemic therapy in SM cT1a RCC cases, including those with bone-only, lung-only, liver-only, and brain-only metastasis, were associated with improved survival outcomes. Additionally, metastasectomy was significantly associated with improved survival in brain-only SM.

This study demonstrates a declining trend in SM cT1a and pT1a RCC. This decline likely reflects the increased incidental detection of small renal masses during routine examinations and the adoption of earlier surgical interventions, which may help reduce the risk of SM. Despite the rarity of SM in T1a RCC, the prognosis for these patients remains poor, and relevant characteristics are still underexplored. When evaluating patients with both clinical and pathologic staging information available, approximately 75% of SM cT1a RCC patients had pathological upstaging. This high proportion of post-surgical pathological upstaging is likely to make a major contribution to the risk of SM. Furthermore, managing general RCC risk factors, such as smoking cessation, body weight, and blood pressure control, may help further reduce SM in T1a RCC.

One of the key findings of our study is the highest frequency of bone metastasis in T1a RCC, which contrasts with existing data indicating that RCC most commonly metastasizes to the lung [13,14,15,16]. In a population-based study of 11,157 RCC patients, the most common metastatic sites were lung (45.2%), bone (29.5%), lymph nodes (21.8%), liver (20.3%), adrenal (8.9%), and brain (8.1%) [16]. A study of the IMDC across histological subtypes of RCC revealed that the most common site was the lung (70%) for clear cell RCC, while it was lymph nodes for papillary (69%) and chromophobe (51%) RCC. Among these subtypes, a similar proportion of bone metastasis (around 30%) was observed [13]. Notably, our study found that bone-only metastasis (59%) was the most common site of metastasis, occurring almost twice as frequently as lung-only metastasis (35%) in SM cT1aRCC. This aligns with findings from other previous studies on T1a and also T1 RCC [17,18]. Collectively, these significant variations in metastatic patterns of SM in T1a RCC are noteworthy, highlighting the heterogeneous nature of tumor biology and unique tropism in small renal masses. These insights are highly relevant to effective patient management, as they may serve as essential tools for developing tailored multimodal treatment strategies for patients with low tumor burden.

Skeletal metastases in RCC are predominantly osteolytic lesions, likely to cause destructive adverse events by compromising bone integrity, which may require surgical treatment or radiotherapy [19]. In addition to bone destruction, the presence of bone metastases is associated with a negative impact on survival outcomes, as has been shown in previous studies [20,21,22]. Current guidelines indicate bone imaging to assess the whole skeleton in symptomatic or advanced RCC patients [23,24]. However, identifying high-risk patients for metastases in early-stage RCC patients might guide providers for accurate diagnostic workup and treatment strategies to improve survival outcomes. Our study revealed that the presence of SM in cT1a RCC patients was most significantly associated with larger tumor size, higher tumor grade, cN1 status, histology of collecting duct and medullary subtypes, as well as sarcomatoid dedifferentiation (Table 2). In a previous registry study of 60,640 T1 stage RCC patients with SM (*n* = 1425; 2.3%), of whom 66.1% had T1a tumors, several factors were significantly associated with higher SM rates, including increasing age (OR = 1.01), tumor size (OR = 1.04), sarcomatoid differentiation (OR = 7.37), collecting duct histology (OR = 6.58), and tumor grades 3–4 (OR = 2.61–4.62) [17]. Although the current data might be affected by the lack of a comparative evaluation of predictors in synchronous and metachronous metastasis, from a clinical standpoint, these findings may help identify high-risk factors for SM in T1a RCC, emphasizing the need for increased vigilance regarding metastatic potential and the use of appropriate diagnostic tools.

In the era of contemporary targeted and immunotherapy strategies for SM RCC, cytoreductive surgery remains a potential treatment option [25,26]. However, its impact on survival outcomes in early-stage T1a RCC remains unclear. Our study revealed an overall reduction in mortality risk for patients who underwent surgical removal of the primary tumor, along with granular differences between bone-only, lung-only, liver-only, and brain-only SM in cT1a RCC (Table 3 and Table 4). The most notable finding was the lower mortality risk in cT1a bone-only SM with partial nephrectomy. While patients with liver-only SM had worse mortality with nephrectomy, lung-only and brain-only SM were associated with better survival outcomes in both nephrectomy and partial nephrectomy groups. Heng et al. reported a significant survival benefit with median OS for those who underwent cytoreductive nephrectomy compared to those who did not (20.6 vs. 9.5 months, *p* < 0.0001) while emphasizing the importance of careful patient selection. However, there was no subgroup analysis based on tumor stage [27]. Given the majority of patients with high tumor burden and advanced local tumor stage in the CARMENA trial, the survival results may have been biased against cytoreductive surgery followed by sunitinib, potentially underestimating the benefit in early-stage tumors that are likely to respond to systemic therapy [8]. As immunotherapy (IO)-based combination treatments represent the current standard of care for patients with synchronous metastatic disease, trials such as SWOG S1931 (PROBE) [28], which focuses on cytoreductive nephrectomy with or without IO-based frontline treatment and the phase II SAMURAI trial [29], which examines stereotactic ablative radiation therapy (SABR) in patients receiving immunotherapy, are crucial for understanding the complex role of primary tumor treatment in improving patient outcomes. These studies could provide critical insights into optimizing treatment strategies in the SM RCC population.

Metastasectomy has had a longstanding role in the treatment of RCC. The main purpose of metastasectomy in RCC patients is to fully resect oligometastatic sites of disease and potentially offer long-term durability with maximal metastasis-directed therapy. Several studies and meta-analyses in overall RCC cohorts have highlighted key factors associated with the survival benefit of metastasectomy, such as the ability to achieve a complete resection of metastases and appropriate patient selection [30,31]. Our study provides insights into the impact of metastasectomy on mortality risk across different metastatic sites in cT1a RCC. In this study, metastasectomy was associated with improved survival in patients with brain-only metastases (HR = 0.26) but did not show the same benefit in patients with bone-only, lung-only, or liver-only metastases. These findings contrast with studies encompassing all tumor stages in RCC, which demonstrate improved survival following metastasectomy, especially in patients with lung metastases [31]. However, our study was limited by the absence of granular data regarding the complete resection of all metastases. In the era of individualized treatment strategies, along with systemic and metastasis-directed therapies, our findings suggest that resecting brain metastases may improve survival in patients with brain-only SM in cT1a RCC. Multimodal approaches should be closely discussed for each patient in multidisciplinary cancer centers.

Our study has several limitations. Given that NCDB utilized hospital-based registry data, we are unable to estimate cancer incidence. Although NCDB does not collect data on cancer-specific mortality or other-cause mortality, it is able to estimate all-cause mortality. In addition, whether or not there was a recurrence of disease after treatment of primary, non-metastatic tumors is not reported in NCDB, which precludes assessment of metachronous disease or recurrence-free survival. Follow-up for cT1a RCC with synchronous metastasis ranged from 0 to 181 months, allowing for computation of 5-year survival (Supplementary Figure S3). While this follow-up was limited, it likely reflects the nature of the disease. Given the rarity of this condition, incorporating additional data from other datasets may not significantly improve the power of this analysis. Moreover, the analysis involves multiple statistical tests, which increases the risk of false positive findings. Although efforts were made to interpret results cautiously, adjustments for multiple testing were not explicitly applied, which may impact the robustness of the findings. NCDB does not report whether the tumor was biopsied prior to initiation of treatment, which may have biased one treatment paradigm over another. Additionally, NCDB does not report postoperative complications, which may impact the mortality rates in RCC patients. Given the retrospective nature of the data, selection bias, as well as physician biases in treatment selection, lack of central pathology review, subtle variations in tumor imaging characteristics, or incomplete accounting of systemic therapies—including treatments received at non-NCDB institutions like smaller community hospitals—and the absence of detailed information on radiotherapy, must be carefully considered when interpreting our results. Despite these limitations, it should be noted that NCDB captures 70% of new cancer diagnoses in the United States, making it the largest cancer registry in the world and thus sufficiently powered to assess trends and outcomes in rare cases of T1a RCC with synchronous metastasis.

## 5. Conclusions

Synchronous metastasis in T1a RCC is most frequently found in bone, followed by lung, brain, and liver. In cases of low tumor burden, such as T1a RCC with isolated metastases, primary tumor resection may provide a survival benefit. Further studies to optimize treatment strategies for patients with synchronous metastatic small renal masses are warranted.

## Figures and Tables

**Table 1:** Multivariable logistic regression models of variables associated with synchronous metastasis in cT1a and pT1a renal cell carcinoma (RCC).

	cT1a RCC with Synchronous Metastasis			pT1a RCC with Synchronous Metastasis		
Variable	OR	95% CI	*p*-Value	OR	95% CI	*p*-Value
Age	1.02	1.02, 1.03	<0.001	1.01	1.01, 1.02	0.002
Year of diagnosis						
2004–2009	—	—		—	—	
2010–2015	1.00	0.86, 1.16	1.000	0.75	0.57, 0.99	0.042
2016–2019	1.02	0.89, 1.18	1.000	0.58	0.44, 0.76	<0.001
Sex						
Female	—	—		—	—	
Male	1.49	1.34, 1.65	<0.001	1.61	1.30, 2.00	<0.001
Race						
White	—	—		—	—	
Black	0.99	0.85, 1.15	1.000	0.78	0.53, 1.11	0.715
Native American	0.83	0.37, 1.64	1.000	0.35	0.02, 1.67	0.925
Asian/Pacific Islander	1.00	0.67, 1.44	1.000	0.98	0.46, 1.81	0.947
Other/Unknown	0.92	0.61, 1.35	1.000	0.75	0.32, 1.49	0.925
Hispanic						
No	—	—		—	—	
Yes	0.83	0.67, 1.03	0.209	1.03	0.68, 1.51	1.000
Unclassified	0.87	0.66, 1.13	0.306	0.97	0.59, 1.53	1.000
Facility Location						
New England	—	—		—	—	
Middle Atlantic	1.09	0.83, 1.44	1.000	1.37	0.82, 2.45	1.000
South Atlantic	1.13	0.87, 1.48	1.000	1.16	0.69, 2.08	1.000
East North Central	1.34	1.04, 1.76	0.257	1.48	0.88, 2.64	1.000
East South Central	1.32	0.99, 1.78	0.507	1.53	0.85, 2.85	1.000
West North Central	1.24	0.92, 1.67	0.954	1.38	0.77, 2.58	1.000
West South Central	1.15	0.86, 1.54	1.000	1.30	0.73, 2.41	1.000
Mountain	1.20	0.85, 1.69	1.000	2.14	1.13, 4.14	0.165
Pacific	1.12	0.84, 1.49	1.000	1.20	0.68, 2.22	1.000
Unclassified	0.68	0.41, 1.10	0.869	0.10	0.02, 0.40	0.034
Facility Type						
Community	—	—		—	—	
Community Comprehensive	0.83	0.68, 1.01	0.054	0.75	0.48, 1.22	0.450
Academic	0.62	0.51, 0.76	<0.001	0.92	0.60, 1.48	0.703
Integrated Cancer Network	0.64	0.52, 0.80	<0.001	0.70	0.44, 1.16	0.450
Median Income						
First Quartile (lowest)	—	—		—	—	
Second Quartile	0.98	0.83, 1.14	0.843	1.16	0.84, 1.62	1.000
Third Quartile	0.94	0.80, 1.10	0.843	0.9	0.64, 1.26	1.000
Fourth Quartile	0.7	0.59, 0.83	<0.001	1.00	0.72, 1.39	1.000
Unclassified	0.83	0.68, 1.00	0.140	0.9	0.59, 1.34	1.000
Charlson Score						
Charlson 0 to 1	—	—		—	—	
Charlson 2 or higher	0.97	0.84, 1.11	0.628	0.69	0.47, 0.98	0.047
Tumor Size	1.70	1.60, 1.82	<0.001	1.79	1.56, 2.06	<0.001
Clinical N Stage						
cN0	—	—		—	—	
cN1	319	237, 436	<0.001	95.3	55.0, 160	<0.001
Unclassified	8.22	6.92, 9.73	<0.001	2.16	1.70, 2.75	<0.001
Histology						
Clear Cell	—	—		—	—	
Papillary	0.39	0.31, 0.49	<0.001	0.40	0.26, 0.58	<0.001
Chromophobe	0.09	0.05, 0.14	<0.001	0.32	0.16, 0.59	0.002
Collecting Duct	11.9	5.24, 25.1	<0.001	—	—	—
Medullary	73.7	20.2, 303	<0.001	—	—	—
RCC NOS	1.24	1.10, 1.40	<0.001	1.09	0.86, 1.36	0.477
Other	2.1	1.73, 2.54	<0.001	0.58	0.23, 1.23	0.409
Sarcomatoid dedifferentiation	2.41	1.69, 3.37	<0.001	5.41	3.24, 8.77	<0.001
Tumor Grade						
Grade 1	—	—		—	—	
Grade 2	1.09	0.80, 1.53	0.593	1.73	1.13, 2.79	0.017
Grade 3	4.08	2.99, 5.68	<0.001	5.09	3.31, 8.23	<0.001
Grade 4	12.2	8.15, 18.3	<0.001	16.7	10.0, 28.7	<0.001
Unclassified	17	12.8, 23.2	<0.001	2.99	1.73, 5.25	<0.001

RCC = renal cell carcinoma, OR = odds ratio, CI = confidence interval, NOS = not otherwise specified.

**Table 2:** Multivariable Cox proportional hazards regression models showing demographic, clinical, pathologic, and treatment variables associated with all-cause mortality in cT1a and pT1a renal cell carcinoma (RCC) presenting with synchronous metastasis.

	cT1a RCC with Synchronous Metastases			pT1a RCC with Synchronous Metastases		
Variables	HR	95% CI	*p*-Value	HR	95% CI	*p*-Value
Age	1.01	1.01, 1.02	<0.001	1.03	1.01, 1.06	0.005
Sex						
Female	—	—		—	—	
Male	1.06	0.92, 1.22	0.402	1.07	0.67, 1.71	0.774
Race						
White	—	—		—	—	
Black	0.86	0.71, 1.05	0.447	1.28	0.53, 3.10	1.000
Native American	2.21	0.88, 5.56	0.376			
Asian/Pacific Islander	0.94	0.59, 1.51	0.802	1.51	0.41, 5.52	1.000
Other/Unknown	0.79	0.46, 1.37	0.802	0.84	0.21, 3.32	1.000
Hispanic						
No	—	—		—	—	
Yes	0.85	0.63, 1.15	0.584	0.71	0.27, 1.88	0.990
Unclassified	1.20	0.70, 2.06	0.584	1.51	0.28, 7.99	0.990
Facility Location						
New England	—	—		—	—	
Middle Atlantic	0.76	0.54, 1.06	0.875	0.37	0.11, 1.17	0.721
South Atlantic	0.87	0.63, 1.20	1.000	0.41	0.13, 1.36	1.000
East North Central	1.28	0.93, 1.77	0.918	0.61	0.20, 1.80	1.000
East South Central	1.11	0.78, 1.60	1.000	0.83	0.25, 2.76	1.000
West North Central	1.25	0.87, 1.81	1.000	0.47	0.15, 1.46	1.000
West South Central	0.95	0.66, 1.37	1.000	0.46	0.12, 1.75	1.000
Mountain	1.49	0.96, 2.32	0.663	0.47	0.11, 1.95	1.000
Pacific	0.96	0.68, 1.36	1.000	0.32	0.09, 1.11	0.648
Unclassified	0.75	0.38, 1.47	1.000	2.38	0.14, 39.5	1.000
Facility Type						
Community	—	—		—	—	
Community Comprehensive	0.88	0.69, 1.11	0.279	2.59	0.71, 9.41	0.255
Academic	0.66	0.51, 0.84	0.003	2.75	0.75, 10.1	0.255
Integrated Cancer Network	0.76	0.58, 1.00	0.095	3.49	0.88, 13.7	0.223
Median Income						
First Quartile (lowest)	—	—		—	—	
Second Quartile	1.02	0.84, 1.25	1.000	0.71	0.32, 1.59	1.000
Third Quartile	1.03	0.85, 1.25	1.000	0.91	0.43, 1.93	1.000
Fourth Quartile	0.96	0.77, 1.18	1.000	1.07	0.51, 2.22	1.000
Unclassified	0.97	0.76, 1.23	1.000	0.39	0.15, 1.00	0.198
Charlson Score						
Charlson 0 to 1	—	—		—	—	
Charlson 2 or higher	1.28	1.08, 1.51	0.004	1.73	0.92, 3.25	0.09
Tumor Size	1.04	0.96, 1.13	0.325	0.8	0.55, 1.14	0.218
cN Stage						
cN0	—	—		—	—	
cN1	1.61	1.37, 1.90	<0.001	3.37	1.32, 8.56	0.021
Unclassified	0.84	0.65, 1.09	0.196	1.31	0.72, 2.40	0.379
Synchronous Metastatic Site *						
Lung	—	—		—	—	
Bone	1.08	0.90, 1.31	0.812	1.36	0.72, 2.56	0.537
Liver	1.24	0.91, 1.68	0.504	3.64	0.84, 15.8	0.338
Brain	1.02	0.74, 1.42	0.890	0.23	0.03, 1.96	0.537
Multiple Organs	1.77	1.46, 2.16	<0.001	1.67	0.71, 3.93	0.537
Histology						
Clear Cell	—	—		—	—	
Papillary	1.00	0.74, 1.35	1.000	0.66	0.28, 1.56	1.000
Chromophobe	1.45	0.45, 4.71	1.000	0.51	0.06, 4.09	1.000
Collecting Duct	1.02	0.52, 2.01	1.000			
Medullary	2.04	0.71, 5.88	0.739			
RCC NOS	1.29	1.11, 1.51	0.006	1.24	0.74, 2.09	1.000
Other	1.86	1.48, 2.34	<0.001			
Sarcomatoid Dedifferentiation	2.04	1.37, 3.06	<0.001	2.03	0.75, 5.49	0.165
Tumor Grade						
1	—	—		—	—	
2	1.38	0.76, 2.51	0.580	2.56	0.59, 11.1	0.662
3	1.63	0.92, 2.91	0.379	2.36	0.58, 9.59	0.662
4	1.15	0.59, 2.26	0.681	1.84	0.34, 10.1	0.662
Unclassified	1.44	0.84, 2.47	0.562	3.46	0.60, 20.0	0.662
Surgery of Primary Site						
Nephrectomy	—	—		—	—	
No Surgery of Primary Site	2.04	1.06, 3.89	0.127			
Cryosurgery/Thermal Ablation	0.76	0.38, 1.53	0.866			
Partial Nephrectomy	0.74	0.48, 1.13	0.482	1.35	0.85, 2.16	0.208
Unclassified/Other	1.66	0.47, 5.89	0.866			
Margin Status						
Negative	—	—		—	—	
Positive	2.07	1.16, 3.70	0.028	2.21	0.86, 5.67	0.198
Unclassified/Not Applicable	1.22	0.64, 2.32	0.546	0.88	0.09, 8.37	0.910
Metastasectomy						
No	—	—		—	—	
Yes	0.83	0.69, 1.00	0.110	0.94	0.57, 1.54	0.805
Unclassified	0.87	0.27, 2.86	0.823			
Systemic Therapy						
No	—	—		—	—	
Yes	0.49	0.42, 0.56	<0.001	1.57	1.00, 2.47	0.097
Unclassified	0.42	0.25, 0.71	0.001	1.45	0.39, 5.33	0.576

RCC = renal cell carcinoma, HR = hazard ratio, CI = confidence interval, NOS = not otherwise specified. * Based on available data from 2010 to 2019.

**Table 3:** Multivariable Cox proportional hazard regression models showing demographic, clinical, pathologic, and treatment variables associated with all-cause mortality in cT1a RCC with synchronous metastasis to lung and bone from 2010 to 2019.

	cT1a Synchronous Metastases to Lung			cT1a Synchronous Metastases to Bone		
Variables	HR	95% CI	*p*-Value	HR	95% CI	*p*-Value
Age	0.98	0.96, 1.01	0.178	1.03	1.02, 1.04	<0.001
Sex						
Female	—	—		—	—	
Male	0.59	0.37, 0.95	0.029	0.97	0.74, 1.27	0.814
Race						
White	—	—		—	—	
Black	0.50	0.25, 0.99	0.192	0.70	0.45, 1.09	0.471
Native American	2.65	0.71, 9.94	0.443	3.83	0.39, 37.5	0.745
Asian/Pacific Islander	1.69	0.29, 9.65	1.000	1.73	0.65, 4.61	0.745
Other/Unknown	0.76	0.06, 9.61	1.000	0.90	0.37, 2.14	0.806
Hispanic						
No	—	—		—	—	
Yes	2.59	0.97, 6.95	0.117	1.05	0.59, 1.86	0.863
Unclassified	2.27	0.34, 15.2	0.398	1.84	0.81, 4.20	0.292
Facility Location						
New England	—	—		—	—	
Middle Atlantic	1.01	0.36, 2.89	1.000	1.00	0.53, 1.90	1.000
South Atlantic	0.76	0.30, 1.90	1.000	1.09	0.59, 2.01	1.000
East North Central	0.86	0.35, 2.09	1.000	0.97	0.52, 1.82	1.000
East South Central	1.92	0.65, 5.66	1.000	1.83	0.89, 3.78	0.911
West North Central	2.05	0.83, 5.10	1.000	1.06	0.54, 2.08	1.000
West South Central	0.56	0.15, 2.07	1.000	1.26	0.61, 2.60	1.000
Mountain	1.78	0.39, 8.19	1.000	1.33	0.57, 3.13	1.000
Pacific	0.79	0.30, 2.09	1.000	1.31	0.68, 2.51	1.000
Unclassified	0.83	0.17, 4.17	1.000	2.64	0.79, 8.77	0.911
Facility Type						
Community	—	—		—	—	
Community Comprehensive	1.90	0.85, 4.22	0.349	1.19	0.69, 2.07	1.000
Academic	0.62	0.29, 1.33	0.438	0.85	0.48, 1.51	1.000
Integrated Cancer Network	1.50	0.65, 3.49	0.438	1.14	0.63, 2.08	1.000
Median Income						
First Quartile (lowest)	—	—		—	—	
Second Quartile	0.77	0.34, 1.74	1.000	0.83	0.55, 1.26	1.000
Third Quartile	1.21	0.57, 2.55	1.000	0.94	0.63, 1.40	1.000
Fourth Quartile	1.05	0.44, 2.53	1.000	0.75	0.49, 1.14	0.715
Unclassified	0.70	0.27, 1.81	1.000	0.90	0.56, 1.44	1.000
Charlson Score						
Charlson 0 to 1	—	—		—	—	
Charlson 2 or higher	2.37	1.32, 4.26	0.004	1.56	1.08, 2.27	0.019
Tumor Size	1.03	0.78, 1.38	0.816	1.19	1.0, 1.41	0.057
cN Stage						
cN0	—	—		—	—	
cN1	1.13	0.69, 1.84	1.000	1.91	1.36, 2.68	<0.001
Unclassified	1.29	0.53, 3.11	1.000	0.90	0.54, 1.50	0.693
Histology						
Clear Cell	—	—		—	—	
Papillary	1.82	1.04, 3.20	0.183	1.17	0.79, 1.74	1.000
Chromophobe	—	—		0.95	0.21, 4.30	1.000
Collecting Duct	0.85	0.27, 2.73	1.000	1.11	0.50, 2.43	1.000
Medullary	2.84	0.62, 13.0	0.537	10.2	2.60, 40.4	0.004
RCC NOS	3.22	0.70, 14.9	0.537	2.65	1.07, 6.54	0.140
Other	2.43	1.12, 5.28	0.147	3.63	2.15, 6.13	<0.001
Sarcomatoid Dedifferentiation	4.09	1.35, 12.4	0.013	2.05	1.04, 4.05	0.039
Tumor Grade						
1	—	—		—	—	
2	0.58	0.16, 2.08	1.000	4.24	1.14, 15.8	0.125
3	1.44	0.39, 5.34	1.000	3.97	1.07, 14.8	0.125
4	0.95	0.24, 3.79	1.000	1.83	0.45, 7.53	0.401
Unclassified	0.78	0.27, 2.24	1.000	3.34	0.95, 11.8	0.125
Surgery of Primary Site						
No Surgery Primary Site	—	—		—	—	
Cryosurgery/Thermal Ablation	0.47	0.13, 1.78	0.269	0.43	0.21, 0.87	0.056
Nephrectomy	0.08	0.01, 0.72	0.047	0.35	0.14, 0.86	0.056
Partial Nephrectomy	0.02	0.00, 0.31	0.013	0.23	0.09, 0.60	0.011
Unclassified/Other	—	—		0.64	0.08, 4.98	0.665
Margin Status						
Negative	—	—		—	—	
Positive	6.75	1.24, 36.8	0.055	2.57	1.04, 6.38	0.083
Unclassified/Not Applicable	0.63	0.07, 5.71	0.682	1.03	0.44, 2.42	0.937
Metastasectomy						
No	—	—		—	—	
Yes	0.91	0.50, 1.67	0.759	0.81	0.59, 1.10	0.169
Unclassified/Not Applicable	—	—		—	—	
Systemic Therapy						
No	—	—		—	—	
Yes	0.36	0.23, 0.57	<0.001	0.57	0.44, 0.74	<0.001
Unclassified	0.09	0.02, 0.43	0.003	0.78	0.29, 2.09	0.627

RCC = renal cell carcinoma, HR = hazard ratio, CI = confidence interval, NOS = not otherwise specified.

**Table 4:** Multivariable Cox proportional hazards regression models showing demographic, clinical, pathologic, and treatment variables associated with all-cause mortality in cT1a RCC with synchronous metastasis to liver and brain from 2010 to 2019.

	cT1a Synchronous Metastases to Liver			cT1a Synchronous Metastases to Brain		
Variables	HR	95% CI	*p*-Value	HR	95% CI	*p*-Value
Age	1	0.97, 1.03	0.989	0.99	0.96, 1.03	0.748
Sex						
Female	—	—		—	—	
Male	0.80	0.38, 1.67	0.546	1.35	0.62, 2.94	0.455
Race						
White	#			#		
Black						
Native American						
Asian/Pacific Islander						
Other/Unknown						
Hispanic						
No	#			#		
Yes						
Unclassified						
Facility Location						
New England	#			#		
Middle Atlantic						
South Atlantic						
East North Central						
East South Central						
West North Central						
West South Central						
Mountain						
Pacific						
Unclassified						
Facility Type						
Community	#			#		
Community Comprehensive						
Academic						
Integrated Cancer Network						
Median Income						
First Quartile (lowest)	—	—		—	—	
Second Quartile	3.44	1.20, 9.87	0.066	0.53	0.14, 1.99	1.000
Third Quartile	3.47	1.06, 11.4	0.080	0.51	0.16, 1.68	1.000
Fourth Quartile	2.40	0.79, 7.29	0.121	1.15	0.25, 5.29	1.000
Unclassified	9.50	1.98, 45.5	0.020	0.59	0.14, 2.55	1.000
Charlson Score						
Charlson 0 to 1	—	—		—	—	
Charlson 2 or higher	2.14	0.91, 5.03	0.082	2.36	0.90, 6.20	0.082
Tumor Size	1.70	1.14, 2.55	0.010	2.44	1.30, 4.59	0.006
cN Stage						
cN0	—	—		—	—	
cN1	0.58	0.27, 1.25	0.326	1	0.34, 2.92	0.997
Unclassified	0.63	0.13, 3.08	0.566	0.34	0.05, 2.37	0.550
Histology						
Clear Cell	—	—		—	—	
Papillary	0.84	0.39, 1.78	1.000	0.49	0.17, 1.43	0.383
Chromophobe	—	—		—	—	
Collecting Duct	—	—		—	—	
Medullary	8.42	1.67, 42.4	0.029	—	—	
RCC NOS	—	—		—	—	
Other	1.16	0.42, 3.22	1.000	2.14	0.63, 7.21	0.383
Sarcomatoid Dedifferentiation	1.00	0.05, 21.5	0.998	1.52	0.17, 13.9	0.709
Tumor Grade						
1	#			#		#
2						
3						
4						
Unclassified						
Surgery of Primary Site						
No Surgery Primary Site	—	—		—	—	
Cryosurgery/Thermal Ablation	—	—		—	—	
Nephrectomy	0.22	0.06, 0.78	0.039 ^	0.08	0.02, 0.41	0.005 ^^
Partial Nephrectomy	0.13	0.01, 2.35	0.165	0.1	0.01, 0.97	0.047 ^^
Unclassified/Other	—	—		—	—	
Margin Status						
Negative	#			#		
Positive						
Unclassified/Not Applicable						
Metastasectomy						
No	—	—		—	—	
Yes	3.14	0.90, 11.0	0.074	0.26	0.10, 0.68	0.006
Unclassified/Not Applicable	—	—		—	—	
Systemic Therapy						
No	—	—		—	—	
Yes	0.41	0.20, 0.85	0.033	0.22	0.10, 0.48	<0.001
Unclassified	1.32	0.07, 25.8	0.853	—	—	

RCC = renal cell carcinoma, HR = hazard ratio, CI = confidence interval, NOS = not otherwise specified. # Variable removed from model to either simplify model or due to low frequency of positive cases within one or more levels in variable. ^ of the 83 cases with liver metastasis, 7 underwent nephrectomy, 3 partial nephrectomy; *p*-values should be interpreted with caution. ^^ of the 90 cases with brain metastasis, 9 underwent nephrectomy, 3 partial nephrectomy; *p*-values should be interpreted with caution.

## Data Availability

Data are available upon reasonable request.

## Supplementary Materials

The following supporting information can be downloaded at: https://www.mdpi.com/article/10.3390/cancers17030364/s1.

## References

[1] Kane C.J., Mallin K., Ritchey J., Cooperberg M.R., Carroll P.R. (2008). Renal Cell Cancer Stage Migration: Analysis of the National Cancer Data Base. Cancer.

[2] American Cancer Society (2024). Cancer Facts & Figures 2024.

[3] Ko J.J., Xie W., Kroeger N., Lee J.-L., I Rini B., Knox J.J., Bjarnason G.A., Srinivas S., Pal S.K., Yuasa T. (2015). The International Metastatic Renal Cell Carcinoma Database Consortium Model as a Prognostic Tool in Patients with Metastatic Renal Cell Carcinoma Previously Treated with First-Line Targeted Therapy: A Population-Based Study. Lancet Oncol..

[4] Tannir N., Albigès L., McDermott D., Burotto M., Choueiri T., Hammers H., Barthélémy P., Plimack E., Porta C., George S. (2024). Nivolumab Plus Ipilimumab versus Sunitinib for First-Line Treatment of Advanced Renal Cell Carcinoma: Extended 8-Year Follow-Up Results of Efficacy and Safety from the Phase III CheckMate 214 Trial. Ann. Oncol..

[5] Frank I., Blute M.L., Leibovich B.C., Cheville J.C., Lohse C.M., Zincke H. (2005). Independent Validation of the 2002 American Joint Committee on Cancer Primary Tumor Classification for Renal Cell Carcinoma Using a Large, Single Institution Cohort. J. Urol..

[6] Nguyen M.M., Gill I.S. (2009). Effect of Renal Cancer Size on the Prevalence of Metastasis at Diagnosis and Mortality. J. Urol..

[7] Renner A., Samtani S., Marín A., Burotto M. (2019). Is Cytoreductive Nephrectomy Still a Standard of Care in Metastatic Renal Cell Carcinoma?. J. Kidney Cancer VHL.

[8] Méjean A., Ravaud A., Thezenas S., Colas S., Beauval J.-B., Bensalah K., Geoffrois L., Thiery-Vuillemin A., Cormier L., Lang H. (2018). Sunitinib Alone or after Nephrectomy in Metastatic Renal-Cell Carcinoma. N. Engl. J. Med..

[9] Bex A., Mulders P., Jewett M., Wagstaff J., Van Thienen J.V., Blank C.U., Van Velthoven R., Del Pilar Laguna M., Wood L., Van Melick H.H.E. (2019). Comparison of Immediate vs Deferred Cytoreductive Nephrectomy in Patients with Synchronous Metastatic Renal Cell Carcinoma Receiving Sunitinib: The SURTIME Randomized Clinical Trial. JAMA Oncol..

[10] Lerro C.C., Robbins A.S., Phillips J.L., Stewart A.K. (2013). Comparison of Cases Captured in the National Cancer Data Base with Those in Population-Based Central Cancer Registries. Ann. Surg. Oncol..

[11] American College of Surgeons (2020). National Cancer Database Participant User File 2019 Data Dictionary.

[12] American College of Surgeons (2016). Facility Oncology Registry Data Standards (FORDS): Revised for 2016.

[13] Dudani S., de Velasco G., Wells J.C., Gan C.L., Donskov F., Porta C., Fraccon A., Pasini F., Lee J.L., Hansen A. (2021). Evaluation of Clear Cell, Papillary, and Chromophobe Renal Cell Carcinoma Metastasis Sites and Association with Survival. JAMA Netw. Open.

[14] Ullah A., Yasinzai A.Q.K., Sakhalkar O.V., Lee K.T., Khan I., Tareen B., Wali A., Waheed A., Khan J., Andam G. (2024). Demographic Patterns and Clinicopathological Analysis of Sarcomatoid Renal Cell Carcinoma in US Population. Clin. Genitourin. Cancer.

[15] Chandrasekar T., Klaassen Z., Goldberg H., Kulkarni G.S., Hamilton R.J., Fleshner N.E. (2017). Metastatic Renal Cell Carcinoma: Patterns and Predictors of Metastases—A Contemporary Population-Based Series. Urol. Oncol..

[16] Bianchi M., Sun M., Jeldres C., Shariat S.F., Trinh Q.-D., Briganti A., Tian Z., Schmitges J., Graefen M., Perrotte P. (2012). Distribution of Metastatic Sites in Renal Cell Carcinoma: A Population-Based Analysis. Ann. Oncol..

[17] Pecoraro A., Palumbo C., Knipper S., Mistretta F.A., Rosiello G., Tian Z., St-Hilaire P.-A., Shariat S.F., Saad F., Lavallée L. (2021). Synchronous Metastasis Rates in T1 Renal Cell Carcinoma: A Surveillance, Epidemiology, and End Results Database-Based Study. Eur. Urol. Focus.

[18] Takayama T., Sugiyama T., Kai F., Suzuki T., Nagata M., Imanishi T., Mizuno T., Sato S., Furuse H., Mugiya S. (2011). Characteristics of Aggressive Variants in T1a Renal Cell Carcinoma. J. Cancer Res. Clin. Oncol..

[19] Grünwald V., Eberhardt B., Bex A., Flörcken A., Gauler T., Derlin T., Panzica M., Dürr H.R., Grötz K.A., Giles R.H. (2018). An Interdisciplinary Consensus on the Management of Bone Metastases from Renal Cell Carcinoma. Nat. Rev. Urol..

[20] McKay R.R., Kroeger N., Xie W., Lee J.-L., Knox J.J., Bjarnason G.A., MacKenzie M.J., Wood L., Srinivas S., Vaishampayan U.N. (2014). Impact of Bone and Liver Metastases on Patients with Renal Cell Carcinoma Treated with Targeted Therapy. Eur. Urol..

[21] Santoni M., Conti A., Procopio G., Porta C., Ibrahim T., Barni S., Guida F.M., Fontana A., Berruti A., Berardi R. (2015). Bone Metastases in Patients with Metastatic Renal Cell Carcinoma: Are They Always Associated with Poor Prognosis?. J. Exp. Clin. Cancer Res..

[22] Beuselinck B., Oudard S., Rixe O., Wolter P., Blesius A., Ayllon J., Elaidi R., Schöffski P., Barrascout E., Morel A. (2011). Negative Impact of Bone Metastasis on Outcome in Clear-Cell Renal Cell Carcinoma Treated with Sunitinib. Ann. Oncol..

[23] Powles T., Albiges L., Bex A., Comperat E., Grünwald V., Kanesvaran R., Kitamura H., McKay R., Porta C., Procopio G. (2024). Renal Cell Carcinoma: ESMO Clinical Practice Guideline for Diagnosis, Treatment and Follow-Up. Ann. Oncol..

[24] European Association of Urology EAU Guidelines. Proceedings of the EAU Annual Congress.

[25] Singla N., Hutchinson R.C., Ghandour R.A., Freifeld Y., Fang D., Sagalowsky A.I., Lotan Y., Bagrodia A., Margulis V., Hammers H.J. (2020). Improved Survival after Cytoreductive Nephrectomy for Metastatic Renal Cell Carcinoma in the Contemporary Immunotherapy Era: An Analysis of the National Cancer Database. Urol. Oncol..

[26] Zarba M., Fujiwara R., Yuasa T., Koga F., Heng D.Y.C., Takemura K. (2024). Multidisciplinary Systemic and Local Therapies for Metastatic Renal Cell Carcinoma: A Narrative Review. Expert Rev. Anticancer Ther..

[27] Heng D.Y., Wells J.C., Rini B.I., Beuselinck B., Lee J.-L., Knox J.J., Bjarnason G.A., Pal S.K., Kollmannsberger C.K., Yuasa T. (2014). Cytoreductive Nephrectomy in Patients with Synchronous Metastases from Renal Cell Carcinoma: Results from the International Metastatic Renal Cell Carcinoma Database Consortium. Eur. Urol..

[28] Kim H., Tangen C.M., Vaishampayan U., Tripathi A., Patel S.K., Shuch B., Barata P., Tan A., Esfeller L., Lara P.N. (2024). SWOG S1931 (PROBE): Phase III Randomized Trial of Immune Checkpoint Inhibitor (ICI) Combination Regimen with or without Cytoreductive Nephrectomy (CN) in Advanced Renal Cancer [NCT04510597]. Urol. Oncol..

[29] Hall W.A., Karrison T., McGregor B.A., Barata P.C., Nagar H., Tang C., Siva S., Morgan T.M., Lang J.M., Kamran S.C. (2023). NRG-GU012: Randomized Phase II Stereotactic Ablative Radiation Therapy (SABR) for Patients with Metastatic Unresected Renal Cell Carcinoma (RCC) Receiving Immunotherapy (SAMURAI). J. Clin. Oncol..

[30] Zaid H.B., Parker W.P., Safdar N.S., Gershman B., Erwin P.J., Murad M.H., Boorjian S.A., Costello B.A., Thompson R.H., Leibovich B.C. (2017). Outcomes Following Complete Surgical Metastasectomy for Patients with Metastatic Renal Cell Carcinoma: A Systematic Review and Meta-Analysis. J. Urol..

[31] Ouzaid I., Capitanio U., Staehler M., Wood C.G., Leibovich B.C., Ljungberg B., Van Poppel H., Bensalah K. (2019). Surgical Metastasectomy in Renal Cell Carcinoma: A Systematic Review. Eur. Urol. Oncol..

