# Supplementary material for: Characteristics and Outcomes of T1a Renal Cell Carcinoma Presenting with Metastasis
Source: Cancers (Basel). 2025 Jan 23;17(3):364. doi: 10.3390/cancers17030364 (PMC11815727; doi:10.3390/cancers17030364)
Supplement: Supplementary file 1 [file cancers-17-00364-s001.zip › cancers-3377785-supplementary.pdf]

Supplementary Figure S1: A flow diagram illustrating the distribution of patients by tumor stage and metastasis status

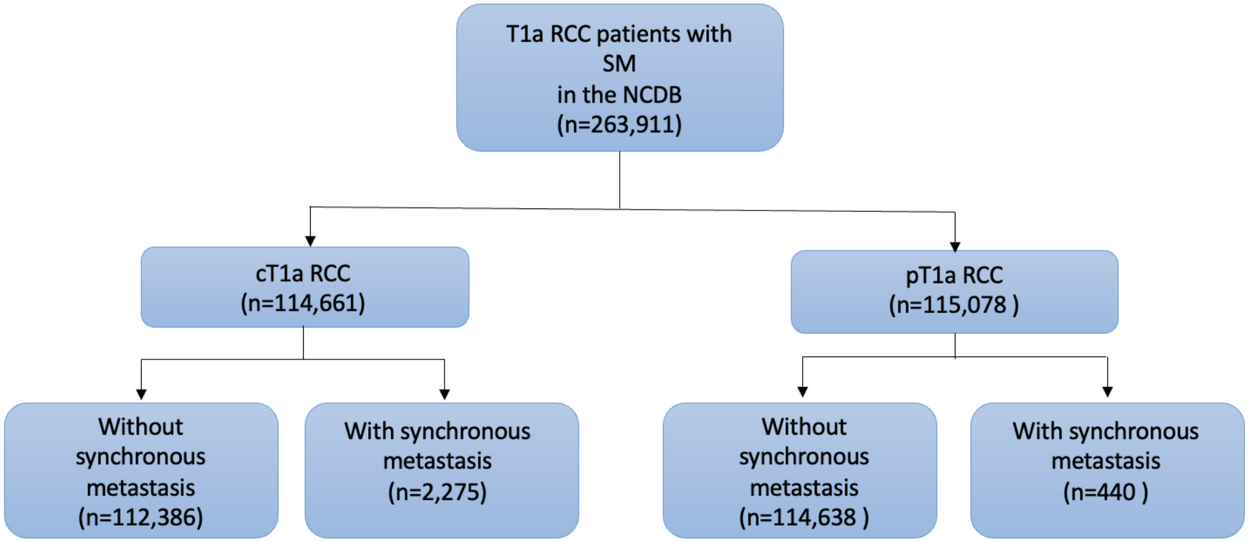

Supplementary Figure S2: Venn diagrams illustrating the overlap of cases between cT1a and pT1a RCC

T1aM0

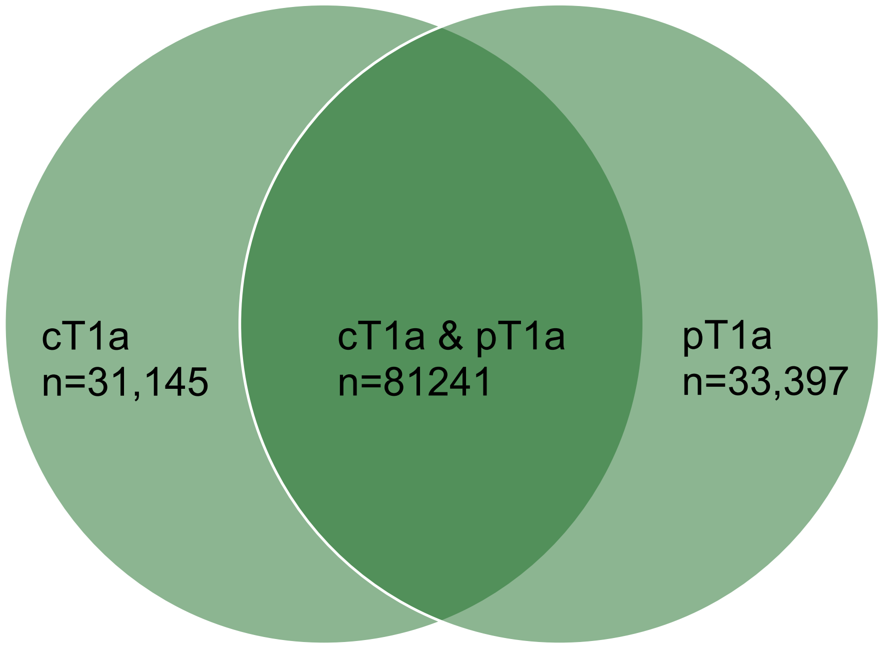

T1aM1

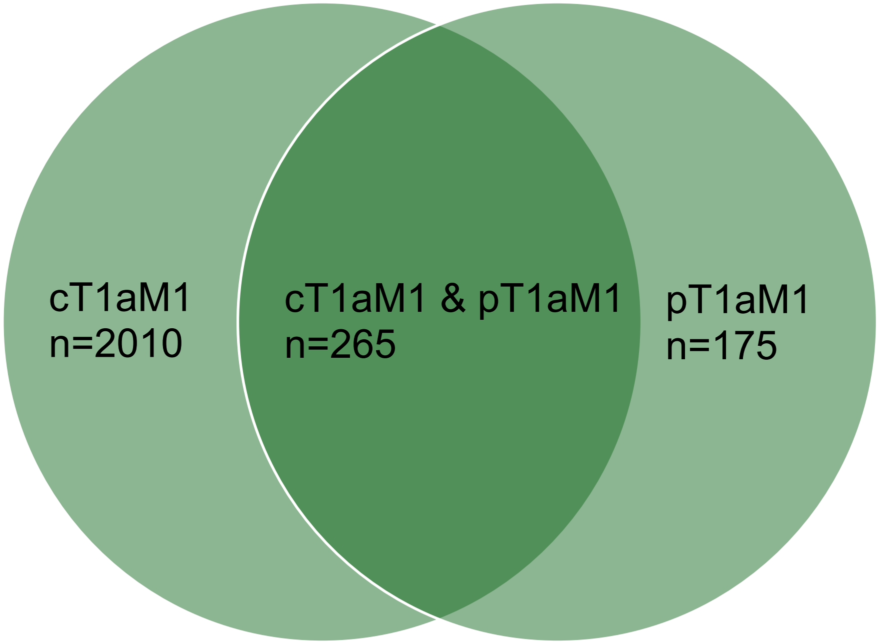

Supplementary Figure S3: Distribution of cT1a patients with synchronous metastasis by follow-up duration in months

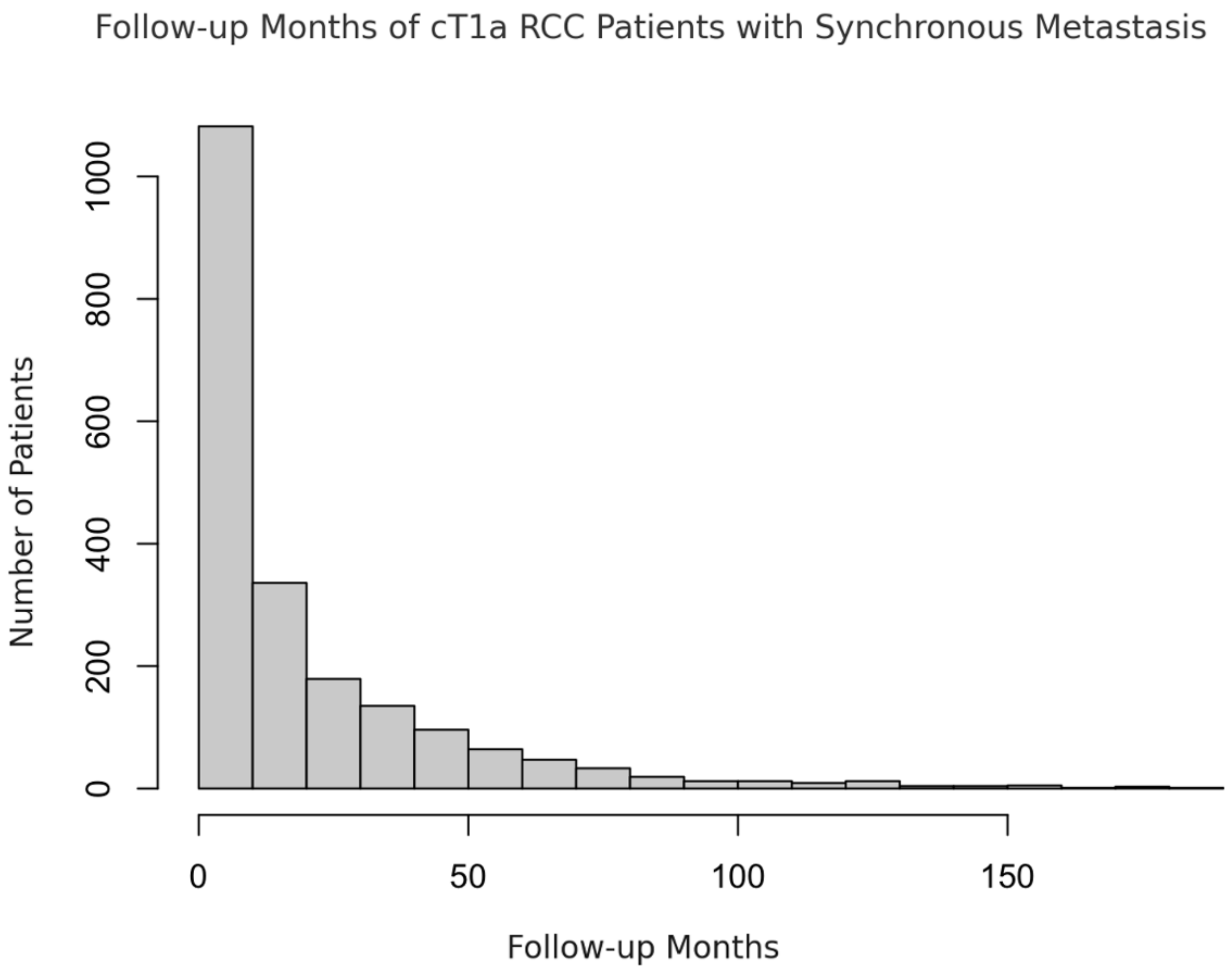

Supplementary Table S1. Demographic, clinical, and pathologic characteristics of cT1a renal cell carcinoma (RCC) without synchronous metastasis, cT1a RCC with synchronous metastasis, pT1a RCC without synchronous metastasis, pT1a RCC with synchronous metastasis.

Variable	cT1a RCC w/o mets, N = 112,386	cT1a RCC w/ synchronous mets, N = 2,275	*p*-value	pT1a RCC w/o mets, N = 114,638	pT1a RCC w/ synchronous mets, N = 440	*p*-value
**Age**	61.00 (51.00, 69.00)	68.00 (60.00, 78.00)	<0.001	59.00 (50.00, 67.00)	62.50 (54.75, 70.00)	<0.001
**Sex**			<0.001			<0.001
Male	65,223 (58%)	1,538 (68%)		66,500 (58%)	313 (71%)	
Female	47,163 (42%)	737 (32%)		48,138 (42%)	127 (29%)	
**Race**			0.028			
White	92,706 (82%)	1,875 (82%)		94,443 (82%)	386 (88%)	
Black	14,024 (12%)	315 (14%)		13,910 (12%)	35 (8.0%)	
Native American	606 (0.5%)	9 (0.4%)		621 (0.5%)	1 (0.2%)	
Asian/Pacific Islander	2,460 (2.2%)	38 (1.7%)		2,762 (2.4%)	10 (2.3%)	
Other/Unknown	2,590 (2.3%)	38 (1.7%)		2,902 (2.5%)	8 (1.8%)	
**Hispanic**			0.002			0.33
No	99,758 (89%)	2,068 (91%)		101,051 (88%)	386 (88%)	
Yes	8,224 (7.3%)	124 (5.5%)		8,821 (7.7%)	30 (6.8%)	
Unknown	4,404 (3.9%)	83 (3.6%)		4,766 (4.2%)	24 (5.5%)	
**Facility Location**			<0.001			<0.001
New England	5,766 (5.1%)	99 (4.4%)		5,853 (5.1%)	17 (3.9%)	
Middle Atlantic	16,736 (15%)	292 (13%)		17,935 (16%)	76 (17%)	
South Atlantic	22,316 (20%)	504 (22%)		21,165 (18%)	78 (18%)	
East North Central	18,853 (17%)	407 (18%)		18,559 (16%)	84 (19%)	

East South Central	8,445 (7.5%)	194 (8.5%)		8,692 (7.6%)	38 (8.6%)	
West North Central	8,298 (7.4%)	187 (8.2%)		8,361 (7.3%)	36 (8.2%)	
West South Central	10,033 (8.9%)	214 (9.4%)		10,213 (8.9%)	42 (9.5%)	
Mountain	3,928 (3.5%)	86 (3.8%)		4,064 (3.5%)	25 (5.7%)	
Pacific	10,380 (9.2%)	240 (11%)		10,610 (9.3%)	42 (9.5%)	
Unknown	7,631 (6.8%)	52 (2.3%)		9,186 (8.0%)	2 (0.5%)	
**Facility Type**			<0.001			<0.001
Community	4,691 (4.2%)	177 (7.8%)		4,533 (4.0%)	25 (5.7%)	
Community Comprehensive	35,652 (32%)	893 (39%)		35,143 (31%)	140 (32%)	
Academic	43,639 (39%)	764 (34%)		44,660 (39%)	199 (45%)	
Integrated Cancer Network	20,773 (18%)	389 (17%)		21,116 (18%)	74 (17%)	
Unknown	7,631 (6.8%)	52 (2.3%)		9,186 (8.0%)	2 (0.5%)	
**Median Income**			<0.001			0.34
First Quartile (lowest)	17,625 (16%)	422 (19%)		17,693 (15%)	66 (15%)	
Second Quartile	22,638 (20%)	524 (23%)		22,775 (20%)	103 (23%)	
Third Quartile	26,561 (24%)	598 (26%)		27,176 (24%)	101 (23%)	
Fourth Quartile	31,761 (28%)	489 (21%)		33,717 (29%)	128 (29%)	
Unknown	13,801 (12%)	242 (11%)		13,277 (12%)	42 (9.5%)	
**Charlson Score**			<0.001			0.024
Charlson 0	76,327 (68%)	1,384 (61%)		79,352 (69%)	293 (67%)	
Charlson 1	23,211 (21%)	523 (23%)		23,783 (21%)	113 (26%)	
Charlson 2	7,674 (6.8%)	202 (8.9%)		7,120 (6.2%)	25 (5.7%)	
Charlson 3 or higher	5,174 (4.6%)	166 (7.3%)		4,383 (3.8%)	9 (2.0%)	
**Tumor Size (cm)**			<0.001			<0.001

1	2,917 (2.6%)	41 (1.8%)		3,266 (2.8%)	6 (1.4%)	
2	30,600 (27%)	326 (14%)		28,526 (25%)	45 (10%)	
3	46,288 (41%)	771 (34%)		44,454 (39%)	120 (27%)	
4	32,581 (29%)	1,137 (50%)		38,392 (33%)	269 (61%)	
**Clinical N Stage**			<0.001			
cN0	109,781 (98%)	1,588 (70%)		97,479 (85%)	282 (64%)	
cN1	75 (<0.1%)	458 (20%)		81 (<0.1%)	26 (5.9%)	
Unknown	2,530 (2.3%)	229 (10%)		17,078 (15%)	132 (30%)	
**Histology**						
Clear Cell	59,007 (53%)	694 (32%)		65,490 (57%)	254 (59%)	
Papillary	15,934 (14%)	127 (5.8%)		16,229 (14%)	29 (6.8%)	
Chromophobe	5,780 (5.2%)	15 (0.7%)		6,501 (5.7%)	10 (2.3%)	
Collecting Duct	44 (<0.1%)	19 (0.9%)		55 (<0.1%)	0 (0%)	
Medullary	4 (<0.1%)	14 (0.6%)		3 (<0.1%)	0 (0%)	
Cyst-Associated	660 (0.6%)	2 (<0.1%)		834 (0.7%)	1 (0.2%)	
RCC NOS	28,785 (26%)	1,114 (51%)		23,921 (21%)	130 (30%)	
Other	2,013 (1.8%)	214 (9.7%)		1,454 (1.3%)	5 (1.2%)	
Unknown	159	76		151	11	
**Sarcomatoid dedifferentiation**	633 (0.6%)	132 (5.8%)	<0.001	518 (0.5%)	39 (8.9%)	<0.001
**Tumor Grade**			<0.001			<0.001
Grade 1	16,184 (14%)	51 (2.2%)		17,705 (15%)	22 (5.0%)	
Grade 2	54,649 (49%)	185 (8.1%)		65,347 (57%)	152 (35%)	
Grade 3	16,545 (15%)	261 (11%)		20,852 (18%)	163 (37%)	
Grade 4	1,297 (1.2%)	90 (4.0%)		1,675 (1.5%)	69 (16%)	

Unknown	23,711 (21%)	1,688 (74%)		9,059 (7.9%)	34 (7.7%)	
**Tumor Necrosis**			<0.001			<0.001
No	47,520 (42%)	219 (9.6%)		54,597 (48%)	148 (34%)	
Yes	2,538 (2.3%)	64 (2.8%)		3,235 (2.8%)	59 (13%)	
Unknown	62,328 (55%)	1,992 (88%)		56,806 (50%)	233 (53%)	
**LVI**			<0.001			<0.001
No	55,921 (50%)	246 (11%)		68,161 (59%)	181 (41%)	
Yes	1,169 (1.0%)	52 (2.3%)		1,446 (1.3%)	26 (5.9%)	
Unknown	55,296 (49%)	1,977 (87%)		45,031 (39%)	233 (53%)	
**Surgery of Primary Site**			<0.001			
No surgery primary site	8,143 (7.2%)	1,841 (81%)		0 (0%)	0 (0%)	
Cryoablation/Thermal Ablation	15,413 (14%)	56 (2.5%)		0 (0%)	0 (0%)	
Nephrectomy	26,256 (23%)	255 (11%)		38,791 (34%)	323 (73%)	
Partial nephrectomy	60,647 (54%)	106 (4.7%)		75,847 (66%)	117 (27%)	
Unknown	1,927 (1.7%)	17 (0.7%)		0 (0%)	0 (0%)	
**Margins**			<0.001			0.078
Negative	84,721 (75%)	331 (15%)		108,290 (94%)	408 (93%)	
Positive	4,602 (4.1%)	35 (1.5%)		5,010 (4.4%)	22 (5.0%)	
Unknown	23,063 (21%)	1,909 (84%)		1,338 (1.2%)	10 (2.3%)	
**Metastasectomy**						
No	112,386 (100%)	1,917 (84%)		114,638 (100%)	296 (67%)	
Yes	0 (0%)	351 (15%)		0 (0%)	142 (32%)	
Unknown	0 (0%)	7 (0.3%)		0 (0%)	2 (0.5%)	
**Systemic Therapy**			<0.001			

No	111,257 (99%)	1,199 (52.7%)		113,279 (99%)	265 (60%)	
Yes	101 (<0.1%)	1,029 (45.2%)		65 (<0.1%)	161 (37%)	
Unknown	1,028 (0.9%)	47 (2.1%)		1,294 (1.1%)	14 (3.2%)	
**Follow-up Months (Median, IQR)**	61.54 (35.98, 97.94)	8.86 (2.63, 27.09)	<0.001	66.96 (39.36, 105.53)	35.32 (16.18, 68.58)	<0.001
Unknown	10,418	221		9,288	20	
**Dead at follow-up**	15,797 (15%)	1,696 (83%)	<0.001	12,884 (12%)	271 (65%)	<0.001
Unknown	10,413	221		9,285	20	
**Synchronous metastasis to lung***						
No		1,126 (63%)			216 (78%)	
Yes		633 (35%)			57 (21%)	
Unknown		29 (1.6%)			4 (1.4%)	
**Synchronous metastasis to bone***						
No		717 (40%)			98 (35%)	
Yes		1,058 (59%)			176 (64%)	
Unknown		13 (0.7%)			3 (1.1%)	
**Synchronous metastasis to liver***						
No		1,491 (83%)			260 (94%)	
Yes		283 (16%)			13 (4.7%)	
Unknown		14 (0.8%)			4 (1.4%)	
**Synchronous metastasis to brain***						
No		1,558 (87%)			258 (93%)	

Yes	211 (12%)	17 (6.1%)
Unknown	19 (1.1%)	2 (0.7%)

IQR = interquartile range, RCC = renal cell carcinoma, w/o = without, w/ = with, mets = metastasis, LVI = lymphovascular invasion, *based on available data from 2010-2019.
